# Genomic and Functional Characterization of the Calcite-Precipitating Bacterium *Bacillus paralicheniformis* ITBMC36: A Promising Agent for Self-Healing Concrete

**DOI:** 10.3390/microorganisms14071437

**Published:** 2026-06-30

**Authors:** Dung Hoang Nguyen, Thanh Mai Luc, Loan Quynh Le, Kien Trung Tran, Ngoc Thi My Tran, Hoang Dang Khoa Do, Ngoc Mach Bao, Danh Hoang Nguyen, Thiet Minh Vu

**Affiliations:** 1 Institute of Life Sciences, Vietnam Academy of Science and Technology, Ho Chi Minh City 700000, Vietnam; nhdung@ils.vast.vn (D.H.N.); ttkien@ils.vast.vn (K.T.T.);; 2 Biotechnology Department, Graduate University of Science and Technology, Vietnam Academy of Science and Technology, Ha Noi City 100000, Vietnam; 3 Functional Genomics Research Center, NTT Hi-Tech Institute, Nguyen Tat Thanh University, Ho Chi Minh City 700000, Vietnamdhdkhoa@ntt.edu.vn (H.D.K.D.);; 4 Nguyen Tat Thanh University Center of Hi-Tech Development, Saigon Hi-Tech Park, Ho Chi Minh City 700000, Vietnam

**Keywords:** *Bacillus paralicheniformis*, microbially induced calcite precipitation, self-healing concrete, whole-genome sequencing, urease, carbonic anhydrase

## Abstract

Cracking is a major cause of concrete deterioration because it allows water and aggressive ions to penetrate the material and accelerate structural damage. Microbially induced calcite precipitation (MICP) has emerged as a promising strategy for crack repair because selected bacteria can precipitate calcium carbonate and thereby seal damaged regions. In this study, we characterized strain ITBMC36, a calcite-precipitating bacterium isolated from a limestone-rich environment in Vietnam, and evaluated its potential for MICP-based crack repair. Strain ITBMC36 produced 26.73 ± 0.81 g/L of mineral precipitate in B4 medium. Mineral characterization showed that the precipitate consisted mainly of calcite, with minor amounts of vaterite and aragonite. Enzymatic assays showed urease activity of 35.71 ± 1.24 U/mL and carbonic anhydrase activity of 1.627 ± 0.010 U/mL. Hybrid genome sequencing generated a complete circular chromosome of 4,410,549 bp, and genome-based taxonomic analysis identified the isolate as *Bacillus paralicheniformis*. Genome mining revealed traits relevant to MICP and survival in cementitious environments, including a complete urea uptake and urease system, five putative carbonic anhydrase genes, exopolysaccharide and biofilm-associated loci, and multiple genes involved in stress response, pH homeostasis, and sporulation. In mortar specimens containing artificial microcracks (0.5 ± 0.1 mm), ITBMC36 promoted progressive crack closure, with visible mineral deposition by day 7 and near-complete sealing by day 35. Together, these results identify *B. paralicheniformis* ITBMC36 as a promising, locally sourced bacterium for MICP-based crack repair and provide a high-quality genome resource for future optimization of bio-based cementitious materials.

## 1. Introduction

Concrete is the most widely used construction material in the world, forming the backbone of modern infrastructure. However, concrete is inherently susceptible to cracking because of shrinkage, thermal stress, mechanical loading, and environmental fluctuation. Even small cracks can reduce durability by allowing water and aggressive ions, such as chlorides and sulfates to penetrate the material and accelerate reinforcement corrosion and chemical deterioration. Conventional repair methods, including epoxy injection or manual patching, are costly, labor-intensive, and temporary, which has increased interest in more sustainable repair strategies.

One promising approach is microbially induced calcite precipitation (MICP), a biomineralization process in which bacteria promote the formation of calcium carbonate. In cementitious systems, this process can help seal cracks and reduce permeability [1,2,3]. Among the mechanisms involved in MICP, ureolysis is one of the most widely studied. In this pathway, bacterial urease hydrolyzes urea to produce ammonia and carbon dioxide (CO_2_) [4]. Ammonia elevates the local pH, shifting carbonate equilibria toward carbonate ions $\left(CO_{3}^{2-}\right),$ which then precipitate with available calcium ions (Ca^2+^). Other important components include carbonic anhydrases (CAs), which catalyze hydration of metabolic CO_2_ to bicarbonate (HCO_3_^−^), and exopolysaccharides (EPS), which form a biofilm matrix that can serve as a nucleation site for calcite crystal growth [5,6].

Because concrete presents a harsh environment characterized by high alkalinity, limited nutrient availability, and mechanical stress, bacteria used for MICP must also be able to survive under challenging conditions. For this reason, research in this field has focused largely on spore-forming or stress-tolerant model organisms. Among them, *Sporosarcina pasteurii* [7], *Bacillus subtilis* [4], and *Bacillus megaterium* [8] are among the best studied. *S. pasteurii* is commonly used as a reference strain because of its strong urease activity and efficient calcite precipitation. By comparison, *Bacillus* species are attractive not only because of their biomineralization capacity, but also because of traits that support survival in cementitious environments, including endospore formation, stress tolerance, and extracellular matrix production. In *B. subtilis*, MICP-related performance has been associated with urease-associated functions and biofilm matrix components that can facilitate crystal nucleation and retention [4], whereas *B. megaterium* has also been reported as an effective calcite-precipitating bacterium with broad metabolic adaptability [8]. Together, these model organisms show that MICP depends on a broader functional toolkit beyond urease alone, including matrix formation, ion regulation, and environmental persistence.

Despite the progress achieved with these organisms, the MICP field still relies heavily on a limited number of model strains [4,9,10,11,12]. Expanding the diversity of candidate bacteria may therefore help identify strains with improved biomineralization performance, greater environmental adaptability, and better suitability for different cementitious systems. In this context, *Bacillus paralicheniformis* is of particular interest. This species is a close relative of *B. licheniformis*, distinguished on the basis of phylogenomic evidence, and is known for its metabolic versatility [13,14]. It has been isolated from diverse environments and explored for applications in agriculture, probiotic development [15], and industrial enzyme production [16]. Although a previous study has reported calcite precipitation in *B. paralicheniformis* [17], its potential for MICP in cementitious materials remains only sparsely investigated, particularly from a genome-resolved perspective.

In this study, we aimed to characterize strain ITBMC36, isolated from carbonate-rich soil and rock in Quang Ninh Province, Vietnam, to assess its suitability for MICP-based crack repair. Specifically, we aim to: (1) quantify its calcite precipitation in vitro; (2) validate its macroscopic crack-healing performance in a standardized cement mortar model; and (3) determine its taxonomic identity using phenotypic and genome-based analyses, generating a complete genome assembly and identifying genetic determinants. By integrating biochemical assays, mineral characterization, genomics, and application testing, this work provides a comprehensive assessment of *B. paralicheniformis* ITBMC36 as a candidate bacterium for sustainable cement-based repair technologies.

## 2. Materials and Methods

### 2.1. Isolation of Calcium Carbonate-Inducing Bacteria

Aggregate samples collected from a limestone quarry in Quang Ninh Province, Vietnam (GPS coordinates: 20°58′05.4″ N 107°10′38.8″ E) were transported to the laboratory for isolation of mineral-forming microorganisms. A modified B4 agar (yeast extract, 4 g/L; dextrose, 5 g/L; calcium acetate, 2.5 g/L; agar, 15 g/L) was used for primary isolation. Single colonies were subcultured in B4 broth and incubated to screen for precipitation activity; strains were considered biomineralizing when visible crystal formation was observed in the broth.

Mineral precipitates were collected by filtration through Whatman No. 1 filter paper, dried at 60 °C to constant weight, and ground into powder. Calcium carbonate formation was verified by acid dissolution/effervescence in hydrochloric acid (HCl). *Sporosarcina pasteurii* DSM33 was obtained from DSMZ German Collection of Microorganisms and Cell Cultures GmbH (Braunschweig, Germany) and used as a reference strain where indicated.

### 2.2. Enzymatic Activity Assays

#### 2.2.1. Preparation of Crude Enzyme Extracts

Cells were cultivated in 50 mL nutrient broth (beef extract, 1 g/L; yeast extract, 2 g/L; peptone, 5 g/L; sodium chloride 5 g/L) until reaching an optical density of OD_600_ = 1. Biomass was harvested by centrifugation (6000 rpm and 4 °C, 10 min) and washed twice with 50 mM HEPES buffer (pH 7.5). Cell disruption was performed by sonication on ice (three pulses of 30 s each). The lysate was clarified by centrifugation (6000 rpm, 4 °C, 10 min) and the resulting supernatant was collected as the crude enzyme extract.

#### 2.2.2. Urease Activity Assay (Phenol–Hypochlorite Method)

Urease activity was measured using the phenol-hypochlorite method. The reaction mixture containing 500 µL crude enzyme extract and 500 µL urease buffer (50 mM HEPES, 25 mM urea) was incubated at 37 °C for 20 min. The reaction was stopped by adding 1.5 mL Solution A (phenol 10 g/L; sodium nitroprusside 50 mg/L) and 1.5 mL Solution B (NaOH 5 mg/mL; NaClO 0.044%). After mixing, samples were incubated at 37 °C for 30 min and absorbance was measured at 620 nm using a microplate reader. Ammonium production was quantified using a standard curve generated from ammonium chloride (NH_4_Cl) solutions (10 µM to 1 mM). Urease activity was expressed as µmol NH_4_^+^ produced per minute under assay conditions [18].

#### 2.2.3. Carbonic Anhydrase Activity Assay

Carbonic anhydrase (CA) activity in crude enzyme extract was measured using the Carbonic Anhydrase Activity Assay Kit (Abcam, Cambridge, UK) according to the manufacturer’s instructions. A nitrophenol standard curve (8–40 nM) was prepared in parallel. CA activity was expressed as nmol nitrophenol released per minute.

### 2.3. Morphological Characterization and Biochemical Tests

Candidate CaCO_3_-producing strains were streaked onto nutrient agar containing beef extract (1 g/L), yeast extract (2 g/L), peptone (5 g/L), sodium chloride (5 g/L), and agar (15 g/L). Colony morphology, including size, shape, color, and texture, was recorded after incubation. Cell morphology was examined by Gram staining and spore staining under a light microscope. Mineral precipitates formed in culture were examined using scanning electron microscopy (SEM).

Metabolic and biochemical properties were assessed using Analytical Profile Index (API) 50 CH and 20 E strips with CH B/E medium (bioMérieux), following the manufacturer’s instructions. Inoculated strips were incubated at 37 °C for 48 h, and reactions were recorded as positive/negative based on recommended color criteria.

### 2.4. Genomic DNA Extraction, Sequencing, and Genome Assembly

Genomic DNA was extracted from a pure culture of ITBMC36 using the EZ-10 Spin Column Soil DNA Miniprep Kit (BioBasic, Markham, ON, Canada). DNA concentration and purity were assessed using a NanoDrop spectrophotometer (Thermo Scientific, Waltham, MA, USA), and integrity was evaluated by agarose gel electrophoresis. DNA concentration was further quantified using the dsDNA BR Qubit assay (Q32850, Invitrogen, Carlsbad, CA, USA) prior to library preparation.

A dual-platform sequencing strategy was employed. Short-read sequencing (2 × 150 bp paired-end) was performed on an Illumina platform by KTest Science Co., Ltd. (Ho Chi Minh City, Vietnam). Long-read sequencing was conducted in-house using an Oxford Nanopore Technologies (ONT) MinION device with an R9.4.1 flow cell (FLO-MIN106, ONT, Oxford, UK). For ONT sequencing, 2 µg of extracted DNA was purified with AMPure XP magnetic bead (A63880, Beckman Coulter, Brea, CA, USA). A library was prepared from 1 µg purified DNA using the NEBNext Ultra II Eng Repair/dA-Tailing Module (E7546, New England Biolabs, Ipswich, MA, USA) and the Ligation Sequencing Kit SQK-LSK109 (ONT, Oxford, UK).

Illumina reads were quality-checked with FastQC (https://www.bioinformatics.babraham.ac.uk/projects/fastqc/) accessed on 14 June 2024 and trimmed using Trimmomatic v.0.39 [19] to remove low-quality bases and adapter sequences. Raw ONT data (FAST5 format) was basecalled with Guppy v.3.1.5 in high-accuracy mode. ONT FASTQ reads were filtered using NanoFilt v.3.8.0 [20] to remove reads with lengths < 1000 bp and reads with mean Q-score < 7. Filtered Illumina and ONT long reads were then used for hybrid assembly with Unicycler v0.5.1 [21]. Assembly completeness and contamination was assessed using CheckM2 [22], and core gene content was evaluated using BUSCO v5 [23] with the Bacillales_odb10 database.

### 2.5. Taxonomic and Phylogenetic Analysis

Whole genome-based taxonomic analyses were conducted. The Type (Strain) Genome Server (TYGS) was used to perform Genome Blast Distance Phylogeny (GBDP) analysis [24]. To confirm species delineation, average nucleotide identity (ANI) and digital DNA-DNA hybridization (dDDH) values were calculated between strain ITBMC36 and its closest relatives using the JspeciesWS online server [25] and Genome to Genome Distance Calculator (GGDC) [26], respectively.

### 2.6. Genome Annotation

The complete genome was first annotated using the Prokaryotic Genome Annotation Pipeline (PGAP; v6.7) [27] for GenBank submission. Functional annotations were further performed using Bakta v1.11.3 [28], Rapid Annotation Using Subsystem Technology (RASTtk) server at https://rast.theseed.org (accessed on 15 December 2025) [29], and eggNOG-mapper v2 for assignment of Clusters of Orthologous Groups (COG) assignment [30]. The final circular genome map was visualized using GenoVi v0.4.3 [31].

All gene identifiers reported in this study correspond to the Bakta annotation (e.g., GMDLPC_000123) and were used consistently for gene localization, operon analysis, and functional interpretation. Gene names and functional descriptions were cross-validated using orthology-based annotation from eggNOG-mapper v2. EggNOG and Bakta annotations were parsed and merged using locus tags as unique identifiers.

### 2.7. Mining for Functional Genes and Comparative Analysis

The annotated genome was manually mined for genes relevant to MICP and fitness in cementitious environment. Keyword-based searches were used to identify genes encoding urease, urea transporters, carbonic anhydrases, EPS biosynthesis proteins, sporulation factors, ion transporters, and stress-response genes. A selected set of MICP-related protein sequences were further examined with InterProScan [32] to identify conserved domains and protein families, providing domain-level validation of key enzymatic and transporter functions. To validate annotations of the urease operon and all candidate CAs, predicted protein sequences were extracted and searched by BLASTp [33] against the UniProtKD/Swiss-Prot database [34]. Secondary metabolite profiles were assessed using antiSMASH v8.0 in the relaxed detection mode [35] and BAGEL4 at http://bagel4.molgenrug.nl/ (accessed on 16 November 2025) [36].

To identify ITBMC36-specific genes, we performed a pangenome analysis using eight publicly available *B. paralicheniformis* genomes selected for high assembly quality and diverse isolation sources. All genomes were re-annotated with Bakta and analyzed with Roary v.3.11.2 [37] to generate a gene presence/absence matrix, which was inspected and visualized using Phandango at https://jameshadfield.github.io/phandango accessed on 20 November 2025 [38]. ITBMC36-specific genes were further analyzed using EggNOG-mapper and InterProScan to assign conserved domains, protein families, and GO/COG terms for functional categorization.

### 2.8. Preparation and Characterization of Bacterially Induced Calcite Precipitation

For mineral precipitation assays, bacteria were cultivated in 50 mL B4 broth at 37 °C under static conditions for 14 days. Culture was centrifuged at 4000 rpm for 15 min to collect the precipitates. The pellet was resuspended in 50 mL TE buffer (pH 8.0) containing lysozyme (1 mg/mL) and incubated at room temperature for 1 h to remove cellular materials. The suspension was centrifuged again (4000 rpm, 15 min) and washed twice with TE buffer. The precipitate was dried at 60 °C for 2 days, and the pellet mass was used as an estimate of precipitate yield, following a published protocol [39].

The morphology of precipitated crystals was examined by field-emission scanning electron microscopy (FESEM; S-4800, Hitachi High-Tech, Hitachinaka, Ibaraki, Japan) operated at an accelerating voltage of 10 kV. Before imaging, precipitates were evenly dispersed and sputter-coated with platinum. Elemental composition was analyzed by energy-dispersive X-ray spectroscopy (EDS) coupled to the FESEM system. Crystal phase was analyzed by X-ray diffraction (XRD; D2 Phaser, Bruker, Karlsruhe, Germany) using CuKα radiation (λ = 1.54184 Å) at 30 kV and 10 mA. Diffraction data were collected using a Lynxeye detector (1D mode, Bruker, Karlsruhe, Germany) with a scan range (2θ) of 20–80°, and a step size of 0.02°.

### 2.9. Concrete Crack-Healing Assay and Water Absorption Test

To evaluate crack-sealing efficiency and changes in water absorption, three experimental groups of mortar specimens were prepared, each comprising ten independent specimens (*n* = 10): (i) uncracked specimens (intact control), (ii) cracked specimens without treatment (cracked control), and (iii) cracked specimens treated with strain ITBMC36 (MICP treatment).

Concrete mortar specimens were prepared by mixing cement (kg), sand (dm^3^), and tap water (L) in a ratio of 1:3:0.6 (*w*:*v*:*v*). The mixture was homogenized, poured into a 90 mm Petri dish, and dried at 60 °C until hardened to generate artificial cracks. To minimize excessive cracking during drying, the specimen surface was periodically sprayed with sterile water. After drying, specimens were immersed in water for 7 days to remove loosely bound material and residual unhydrated cement paste. Baseline crack width was quantified from stereomicroscope images using ImageJ, and cracked specimens with an initial width of 0.5 ± 0.1 mm were selected for subsequent treatment and analysis.

For bacterial treatment, strain ITBMC36 was cultured in nutrient broth at 37 °C and 120 rpm for 24 h, harvested by centrifugation (10,000 rpm, 4 °C, 10 min) and resuspended in B4 broth to an optical density of OD_600_ = 1.0 (approximately 10^8^ CFU/mL). Concrete specimens were incubated at 37 °C. A single 1 mL aliquot of the bacterial suspension was applied directly into each crack at the start of the experiment. Control specimens received 1 mL sterile B4 medium without bacterial inoculation. Crack healing was monitored over 35 days at 6-day intervals. At defined time points, specimens (*n* = 6) were dried and examined under a stereomicroscope to assess mineral deposition and crack closure, following the imaging/assessment procedure described previously [40]. Crack images were recorded during the experiment and processed using ImageJ software v1.53e (NIH, Bethesda, MA, USA). Images were converted to 8-bit grayscale and thresholded to generate binary images for quantitative analysis. Crack area was measured at each time point, and healing efficiency was calculated as the percentage of restored area relative to the initial crack area.

Water absorption was measured using a standard immersion-based protocol [41]. Briefly, uncracked specimens, cracked untreated specimens, and cracked specimens treated with ITBMC36 were oven-dried at 105 °C to constant mass and weighed to obtain dry mass. Specimens were then fully immersed in water, with the water level maintained 10–20 mm above the specimen surface. Specimen mass was recorded after 3, 6, 12, 24, and 48 h of soaking, and water absorption was calculated as the percentage increase in mass relative to the initial dry mass.

### 2.10. Statistical Analysis

All quantitative data are presented as mean ± standard deviation (SD) from at least three independent experiments. Statistical analysis was performed using GraphPad Prism version 9 (GraphPad Software, San Diego, CA, USA). For comparisons between two groups, Student’s *t*-test was used. For time-course datasets (e.g., crack area restoration over time), two-way ANOVA (treatment × time) or repeated-measures two-way ANOVA (when the same specimens were tracked across time) was applied. Differences were considered statistically significant at *p* < 0.05.

## 3. Results

### 3.1. Isolation and Initial Characterization of the Calcite-Precipitating Strain ITBMC36

**Figure 1:**
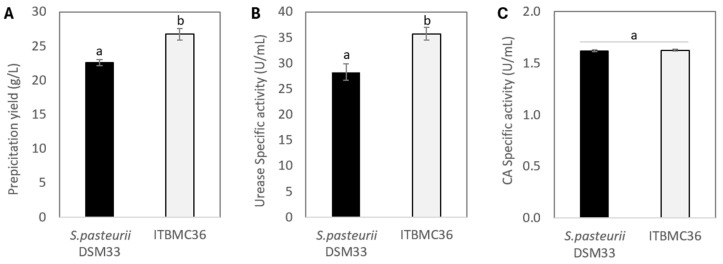
Comparison of precipitation yield and enzyme activities between strains ITBMC36 and *S. pasteurii* DSM33. (**A**), precipitation yield; (**B**), Urease activity; (**C**) Carbonic anhydrase activity. Data are presented as mean ± SD. Within each parameter, bars labeled with different letters are significantly different based on Student’s *t*-test (*p* < 0.05).

Among the bacterial strains isolated from limestone samples, strain ITBMC36 exhibited the strongest biomineralization in B4 medium (Figure 1). The strain produced 26.73 ± 0.81 g/L of mineral precipitate. The recovered material showed vigorous effervescence after treatment with HCl, confirming the presence of carbonate minerals (CaCO_3_).

When compared under the same conditions, the reference strain *Sporosarcina pasteurii* DSM33 produced 22.60 ± 0.81 g/L of precipitate (Figure 1A). Urease and carbonic anhydrase activities were then measured using crude enzyme extracts. Carbonic anhydrase activity did not differ significantly between ITBMC36 and DSM33, whereas urease activity was higher in ITBMC36 (35.71 ± 1.24 U/mL) than in DSM33 (28.27 ± 1.57 U/mL) (Figure 1B,C). Together, these results indicate that ITBMC36 has strong ureolytic activity and a high capacity for mineral precipitation.

**Figure 2:**
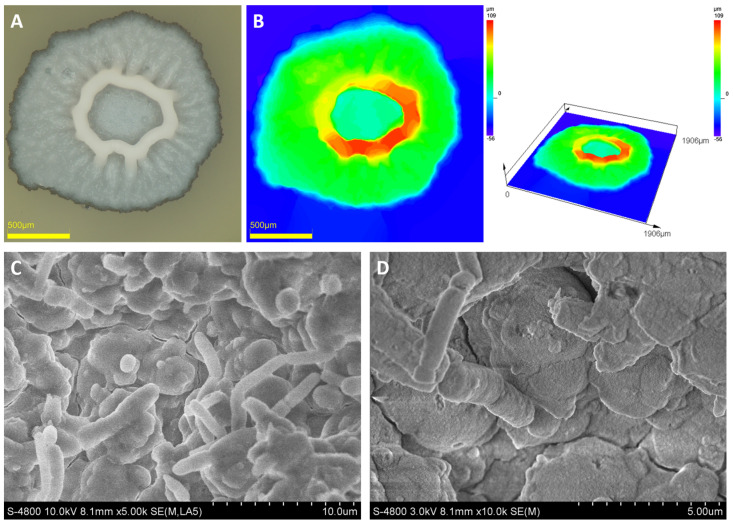
Morphological characteristics of strain ITBMC36. (**A**,**B**) Colony morphology on nutrient agar after 24 h of incubation, shown as a 2D and a 3D height profile. (**C**,**D**) SEM images of the cell pellet containing bacterial cells and mineral crystals at 5000× and 10,000× magnification.

On nutrient agar, colonies of ITBMC36 were opaque white, moist, and circular, with irregular margins and a raised central ring (Figure 2A,B). After 24 h of incubation, the average colony diameter was 1.51 ± 0.19 mm. SEM analysis of cells grown in B4 broth showed rod-shaped bacteria approximately 4.5 ± 0.3 × 0.5 ± 0.1 µm in size (Figure 2C,D). Mineral crystals were observed around the cell pellet, indicating active association between bacterial growth and calcium carbonate formation. Gram staining showed that the strain is Gram-positive, and spore staining confirmed its ability to form endospores.

### 3.2. Mineral Characterization and Crack-Sealing Performance

#### 3.2.1. Mineral Precipitation in Culture

**Figure 3:**
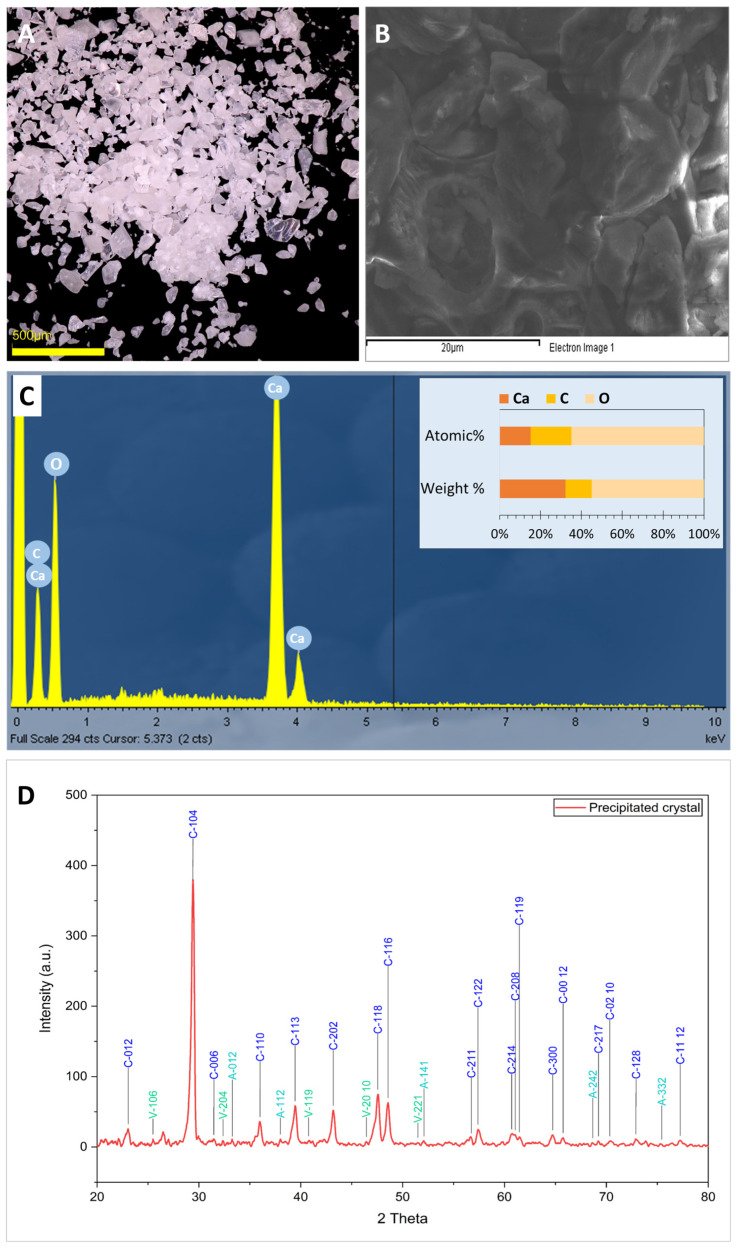
Characterization of mineral crystal precipitated from the culture medium. (**A**) Image of the precipitate after lysozyme treatment. (**B**) SEM micrograph of the precipitated sample. (**C**) EDS spectrum showing major elemental composition (weight % and atomic %); circled labels on peaks indicate identified elements. (**D**) XRD patterns of precipitate samples. Letters A, V, and C denote polymorphs aragonite, vaterite, and calcite, respectively; numbers indicate Miller indices (hkl).

Mineral precipitates produced by ITBMC36 in B4 broth were characterized by SEM-EDS and XRD. EDS analysis showed that carbon, oxygen, and calcium were the dominant elements in the precipitated material, with atomic percentages of 20.00%, 64.82%, and 15.18%, respectively, corresponding to weight percentages of 12.74%, 55.00%, and 32.26%. This composition is consistent with calcium carbonate-rich biominerals (Figure 3A–C).

XRD analysis showed that the precipitates contained multiple calcium carbonate polymorphs (Figure 3D). Peaks corresponding to calcite, vaterite, and aragonite were detected by comparison with reference diffraction patterns (aragonite: RRUFF-R040078; calcite: RRUFF-R050009; vaterite: AMCSD-0019869). Calcite was the dominant crystalline phase, as indicated by the strong reflection for the calcite (104) plane and additional peaks assigned to the (102), (110), (113), (202), (118), and (116) planes. Minor peaks corresponding to vaterite and aragonite were also present. These results indicate that ITBMC36 produces predominantly calcite, with smaller amounts of other CaCO_3_ polymorphs.

#### 3.2.2. Crack-Sealing Activity in Mortar Specimens

**Figure 4:**
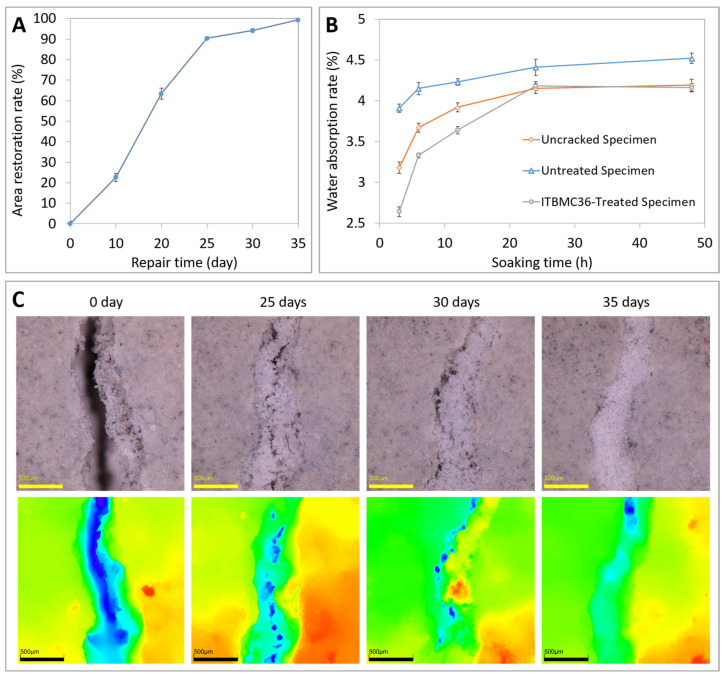
Crack-healing and water absorption performance of ITBMC36–treated concrete specimens. (**A**) Crack area restoration over a 35-day healing period. (**B**) Water absorption rates of the specimens for 48 h immersion. (**C**) Optical and color-mapped surface morphology of the cracked region at 0, 25, 30, and 35 days, illustrating gradual crack closure and surface restoration via microbially induced CaCO_3_ precipitation. The color-mapped image shows height variation, with blue indicating the lowest areas and red the highest. Scale bar = 500 µm.

The crack-sealing performance of ITBMC36 was evaluated using mortar specimens containing artificial microcracks with an average width of 0.5 ± 0.1 mm. In specimens treated with ITBMC36, visible mineral deposition was observed by day 7, the cracks appeared largely sealed by day 25, and complete closure was observed by day 35 (Figure 4A). In contrast, control specimens treated with sterile medium showed no visible crack sealing over the same period. These observations indicate progressive crack filling associated with bacterial treatment.

Water absorption measurements further supported the crack-sealing effect of ITBMC36 (Figure 4B). Untreated cracked specimens showed the highest water absorption throughout the experiment, consistent with rapid water ingress through open cracks. Uncracked specimens showed a moderate and steady increase in water uptake, reflecting the intrinsic pore structure of intact mortar. By comparison, ITBMC36-treated specimens showed reduced water absorption during the early soaking period (3–12 h), indicating that crack filling limited initial water entry. At later time points (24–48 h), water uptake in treated specimens increased and approached that of uncracked specimens as the cement matrix became saturated.

Stereomicroscopy confirmed progressive mineral accumulation within the cracks over time (Figure 4C). Optical images showed gradual narrowing and eventual filling of the crack space, while depth-mapped images showed a marked reduction in deep-blue regions, consistent with loss of crack depth. Together, the imaging and water-absorption data show that ITBMC36 promoted effective crack closure in the mortar model.

**Figure 5:**
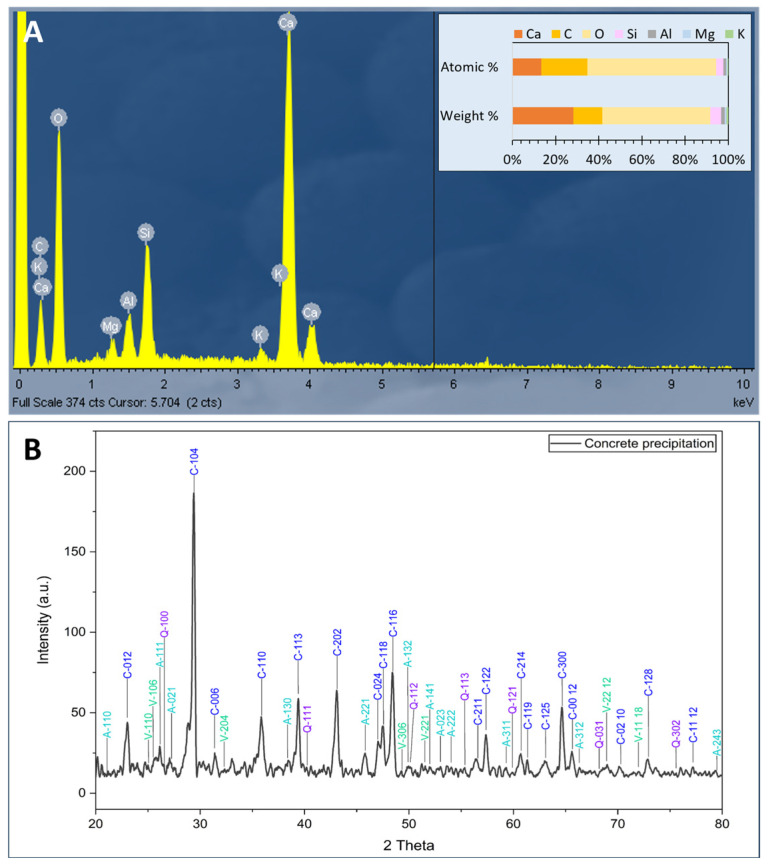
Compositional analysis of mineral crystals collected from cracks in ITBMC36-treated mortar specimens. (**A**) EDS spectrum showing elemental composition, circle labels on the peaks indicate identified elements. (**B**) XRD diffraction patterns; the letters A, V, C, and Q correspond to aragonite, vaterite, calcite, and quartz, respectively; numerical values indicate Miller indices (hkl).

Mineral deposits recovered from treated cracks were further analyzed by EDS and XRD. EDS confirmed that calcium, carbon, and oxygen were the dominant elements, consistent with CaCO_3_-rich material (Figure 5A). Smaller amounts of silicon (Si: 4.99 wt%, 3.39 at%), aluminum (Al: 1.58 wt%, 1.12 at%), magnesium (Mg: 0.89 wt%, 0.70 at%), and potassium (K: 0.85 wt%, 0.41 at%) were also detected, likely reflecting incorporation of components derived from sand and cement (Figure 5A). XRD analysis showed that calcite was the major crystalline phase, with minor amounts of aragonite, vaterite, and quartz (Figure 5B). These results confirm that ITBMC36 treatment led to deposition of calcite-dominant CaCO_3_ within the crack environment.

### 3.3. Metabolic and Biochemical Characteristics of ITBMC36

Biochemical profiling showed that ITBMC36 has broad metabolic capacity (Table 1). The strain utilized a wide range of carbohydrates, including glycerol, L-arabinose, ribose, D-xylose, galactose, D-glucose, D-fructose, D-mannose, mannitol, sorbitol, methyl-D-glucoside, amygdalin, arbutin, esculin, salicin, cellobiose, maltose, lactose, sucrose, trehalose, starch, glycogen, turanose, and tagatose. It was also positive for ONPG, arginine dihydrolase, Voges–Proskauer reaction, gelatinase, nitrate reductase, catalase, and oxidase. In particular, the positive urease reaction is consistent with a ureolysis-based mechanism for calcium carbonate precipitation. Overall, these results showed that ITBMC36 is metabolically versatile and carries several phenotypic traits relevant to biomineralization.

### 3.4. Genome Assembly and Species Identification

**Figure 6:**
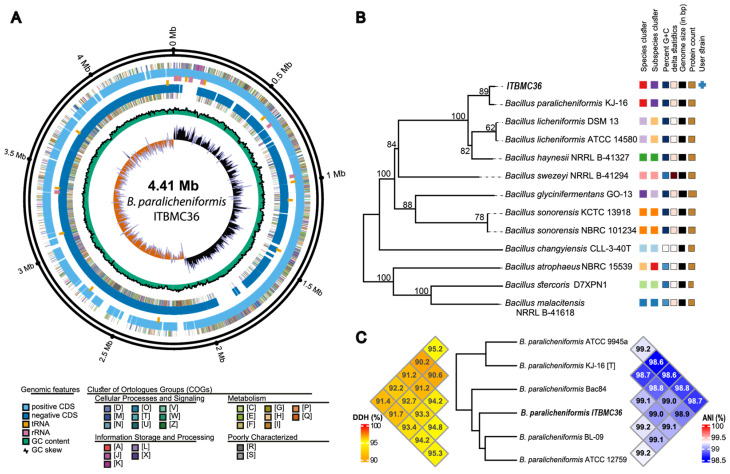
Whole-genome based species delineation of strain ITBMC36. (**A**) Circular map of the *B. paralicheniformis* ITBMC36 genome. Genomic features are color-coded as indicated; predicted CDSs assigned to COG functional categories are shown using the corresponding COG color codes (**B**) Genome Blast Distance Phylogeny (GBDP) tree reconstructed using TYGS, showing the phylogenetic position of strain ITBMC36 (highlighted in bold). The tree is based on whole-genome sequence data and rooted at midpoint. Branch support values are shown at nodes. The colored boxes on the right indicate TYGS species cluster, subspecies cluster, genomic G+C content, delta statistics, protein count, and user strain status, as shown in the panel legend. (**C**) Heatmap of pairwise dDDH and ANI values calculated between ITBMC36 and its closest relatives. The left heatmap shows dDDH values, with the yellow-to-red color scale indicating increasing dDDH percentage values. The right heatmap shows ANI values, with the blue-to-red color scale indicating increasing ANI percentage values.

To place strain ITBMC36 in a robust taxonomic context and to provide a genome resource for interpreting the biomineralization and crack-sealing phenotypes described above, we performed hybrid whole-genome sequencing using Oxford Nanopore and Illumina platforms. Sequencing generated 7.5 Gb of long reads and 6.3 Gb of paired-end short reads (2 × 150 bp). Hybrid assembly with Unicycler produced a single circular chromosome of 4,410,549 bp with a GC content of 45.8% (Table 2; Figure 6A). The assembly showed high quality, with 100% completeness and 0.22% contamination according to CheckM2, and 99.3% of expected single-copy orthologs recovered by BUSCO. These metrics indicate that the genome assembly is suitable for detailed downstream analysis.

Phenotypic and biochemical features initially supported assignment of ITBMC36 to the genus *Bacillus*. Whole-genome analysis using TYGS and GBDP further placed the strain within the *Bacillus paralicheniformis* clade (Figure 6B). Pairwise genome relatedness values supported this classification, with 98.2% ANI and 85.5% dDDH between ITBMC36 and the *B. paralicheniformis* type strain KJ-16 (Figure 6C). Based on these polyphasic data, the isolate was identified as *Bacillus paralicheniformis* ITBMC36.

### 3.5. Genomic Basis of Biomineralization-Related Functions in ITBMC36

#### 3.5.1. Functional Overview of the ITBMC36 Genome

**Figure 7:**
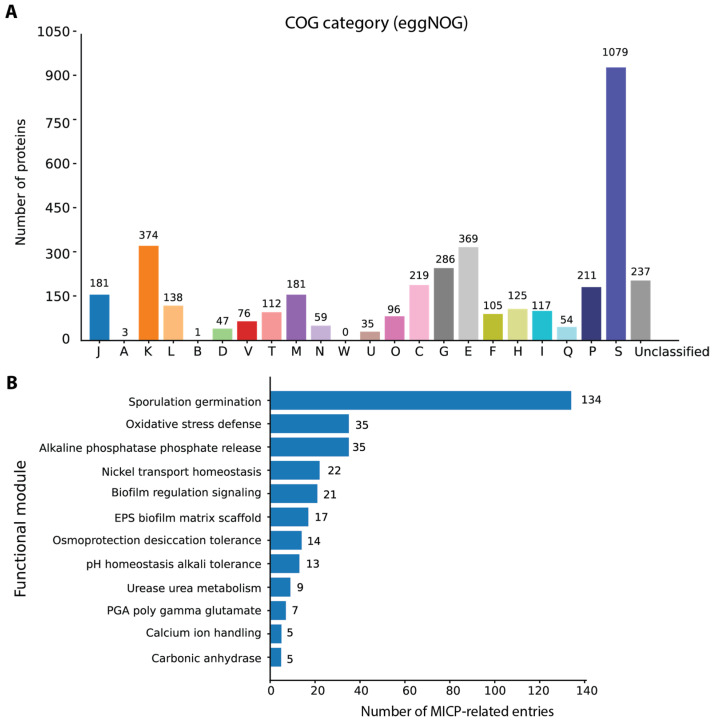
Functional overview and MICP-related genomic features of strain ITBMC36. (**A**) Distribution of predicted proteins across COG functional categories. (**B**) Summary of curated functional modules relevant to biomineralization and environmental persistence. Bars indicate the number of genes assigned to each module based on integrated Bakta/eggNOG-mapper annotation.

Genome annotation identified 4647 protein-coding sequences, 81 tRNA genes, and 24 rRNA genes (Table S1). COG-based functional classification showed that the largest categories were transcription (K; 374 genes), amino acid transport and metabolism (E; 369 genes), and carbohydrate transport and metabolism (G; 286 genes) (Figure 7A). Genes involved in energy production and conversion (C; 219 genes), as well as inorganic ion transport and metabolism (P; 211 genes), were also well represented. These features indicate that ITBMC36 is a metabolically versatile bacterium with substantial regulatory and physiological capacity.

Targeted genome mining identified groups of genes relevant to MICP (Figure 7B; Table 3). The most abundant category comprised sporulation and germination genes (134 genes), consistent with the endospore-forming phenotype of the strain. Additional gene sets were associated with oxidative stress defense (35 genes), alkaline phosphatase activity (35 genes), nickel transport and homeostasis (22 genes), biofilm formation (21 genes), matrix scaffolding (17 genes), osmoprotection (14 genes), and pH homeostasis (13 genes). Core genes related to ureolysis and carbonate generation were also present, supporting the biochemical phenotype observed experimentally.

#### 3.5.2. Urease System and Carbonic Anhydrases

**Figure 8:**
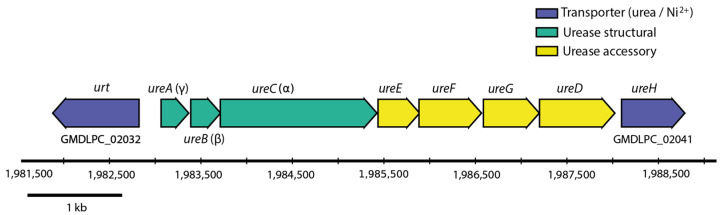
Genomic organization of the urea uptake and urease gene clusters in *B. paralicheniformis* strain ITBMC36.

The genome of ITBMC36 contains a complete, clustered urease operon (Figure 8). This locus includes core genes encoding urease enzyme subunit *ureA* and *ureB*, and the catalytic subunit *ureC*. It also contains accessory genes required for assembly and activation of the metalloenzyme (*ureD*, *ureF*, *ureG*, and the nickel-delivering metallochaperone *ureE*). Flanking genes encode transport functions for substrate and cofactor acquisition, including a urea transporter (*urt*) and a high-affinity nickel transporter (*ureH*). The presence of this complete urease module is consistent with the strong urease activity measured experimentally.

**Figure 9:**
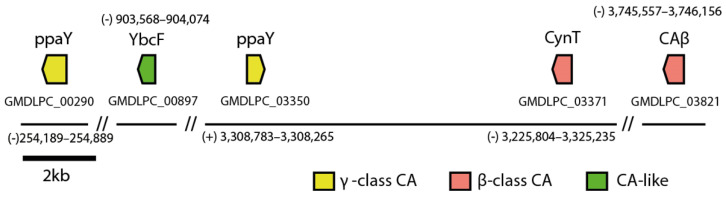
Carbonic anhydrase (CA) genes identified in the genome of *B. paralicheniformis* ITBMC36. The plus (+) and minus (−) signs before the coordinates indicate that the corresponding genes are located on the positive or negative DNA strand, respectively.

In addition to the urease system, the genome encodes five putative carbonic anhydrase (CA) genes that can accelerate interconversion of CO_2_ and bicarbonate (HCO_3_^−^) during mineralization (Figure 9, Table S2). These include two β-class CAs (GMDLPC_03371, GMDLPC_03821) and two γ-class Cas (GMDLPC_00290, GMDLPC_03350) and one additional CA-like gene, *YbcF* (GMDLPC_00897). The presence of multiple carbonic anhydrase candidates suggests functional redundancy and may support efficient interconversion of CO_2_ and bicarbonate during biomineralization. The predicted protein sequences for the urease locus (*UreA*–*UreH*) and all CA candidates were queried by BLASTp against UniProtKB/Swiss-Prot. The top hits consistently supported the automated assignments, matching canonical urease structural/accessory proteins and β-/γ-class carbonic anhydrases (Table S3).

#### 3.5.3. Genes Associated with Mineral Nucleation and Retention

**Figure 10:**
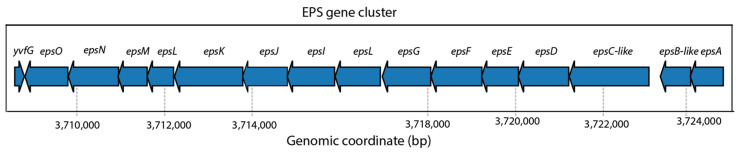
Genomic organization of the EPS biosynthesis/export cluster in ITBMC36.

The genome also contains several loci associated with extracellular matrix production and biofilm formation, which may support localized mineral nucleation and retention. A near-complete EPS biosynthesis and export module was identified, including multiple glycosyltransferases (*epsD*/*epsE*/*epsF*/*epsJ*/*epsL*), polymer modification enzymes (*epsI*, pyruvyl transferase; *epsM*, acetyltransferase), and export-related components (*epsK* and *epsG*) (Figure 10). These genes are consistent with the ability to form extracellular polymeric material that could help create a favorable microenvironment for mineral deposition.

**Figure 11:**
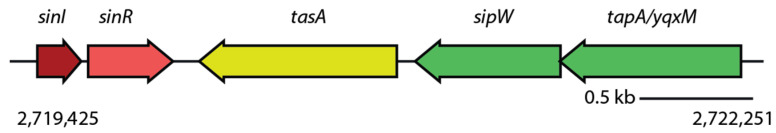
Genomic organization of the biofilm matrix operon in ITBMC36 genome. The *sinI*-*–sinR* regulatory module precedes the *tasA–sipW–tapA* operon, which encodes the amyloid fiber matrix protein TasA, the signal peptidase SipW, and the anchoring protein TapA. Gene orientation and spacing are shown to scale.

ITBMC36 also encodes the conserved *tapA*–*sipW*–*tasA* operon (Figure 11), which is involved in assembly of amyloid-like biofilm fibers. The genome further contains *sinI* (GMDLPC_02738) and *sinR* (GMDLPC_03109), regulators associated with matrix production, as well as *bslA* (GMDLPC_04112) and duplicated *bslB*-like genes (GMDLPC_04214 and GMDLPC_04215) linked to surface properties and biofilm structure. Together, these features suggest that ITBMC36 can form a stable extracellular matrix that may enhance cell retention and surface-associated CaCO_3_ precipitation.

**Figure 12:**
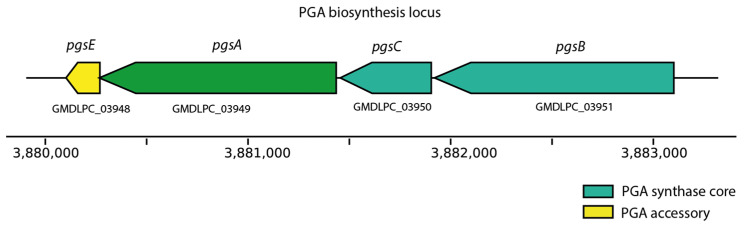
Genomic organization of the γ-PGA biosynthesis locus in *B. paralicheniformis* ITBMC36.

A complete *pgsE*–*pgsA*–*pgsC*–*pgsB* locus for poly-γ-glutamic acid biosynthesis was also identified (Figure 12). Because poly-γ-glutamic acid is an anionic extracellular polymer, it may promote local calcium accumulation near the cell surface and thereby favor heterogeneous nucleation of calcium carbonate. Collectively, the EPS, amyloid fiber, and γ-PGA loci support the idea that ITBMC36 can generate an extracellular scaffold favorable for crack-localized mineral deposition.

Collectively, the presence of EPS, TasA amyloid fibers, and γ-PGA biosynthesis genes suggests that ITBMC36 can produce a chemically reactive extracellular matrix. Such scaffolding is consistent with localized CaCO_3_ deposition and retention within cracks during MICP.

#### 3.5.4. Stress Adaptation, pH Homeostasis, and Sporulation

The ITBMC36 genome contains several features consistent with survival under alkaline and nutrient-limited conditions. Multiple cation/proton antiporters were identified, including three nhaC–type Na^+^/H^+^ antiporters (GMDLPC_00551, GMDLPC_01102, and GMDLPC_04238), *yuiF* (GMDLPC_03538), and a complete seven-subunit mnhA–mnhG (Mrp/Mnh) antiporter system. A putative Ca^2+^/H^+^ exchanger (GMDLPC_00875) was also present. These systems are consistent with a role in pH homeostasis and ion balance during growth in cementitious environments.

Genes involved in sporulation and germination were also abundant (Table 3). The genome contains the master sporulation regulator *spo0A* (GMDLPC_02465), its associated components, *spo0B* (GMDLPC_02848) and *spo0F* (GMDLPC_04118). This initiation is supported by a cascade of sporulation-specific sigma factors that control gene expression in a stage-specific manner, including *sigH* (GMDLPC_00136), *sigF* (GMDLPC_02591), *sigE* (GMDLPC_01792), *sigG* (GMDLPC_01793), and *sigK* (GMDLPC_02875). Endospore development and protective structure was supported by multiple stage-specific genes and coat proteins. These include the *spoIIIA* operon (GMDLPC_02604–02611), which is vital for spore development, and genes like *spoIVA* (GMDLPC_02520) and *spoVM* (GMDLPC_01842) involved in coat and cortex assembly. Outer protection of the spore is further reinforced by spore coat proteins, notably *cotY* (GMDLPC_01343, GMDLPC_01344) and *cotE* (GMDLPC_01965), while *pdaB* (GMDLPC_00195) likely mediates the cortex modifications necessary for thermal and chemical resistance. These features are essential for the survival of the bacteria once embedded within the calcium carbonate matrix produced during biomineralization.

For spore reactivation when conditions improve, the genome encodes germination machinery capable of sensing external cues (Table 3). The gerA receptor complex, comprising *gerAA* (GMDLPC_02259), *gerAB* (GMDLPC_02255, GMDLPC_02258), and *gerAC* (GMDLPC_02256), provides a primary system for detecting nutrient germinant such as L-alanine. This signaling is supported by auxiliary proteins including *gerD* (GMDLPC_00193) and *gerP* cluster (GMDLPC_01226–01231), which can facilitate rapid transition from dormancy to vegetative growth by increasing spore coat permeability. These survival and germination systems collectively indicate the capacity for long-term persistence with reactivation upon water and nutrient availability.

#### 3.5.5. Biosynthetic Gene Clusters

**Figure 13:**
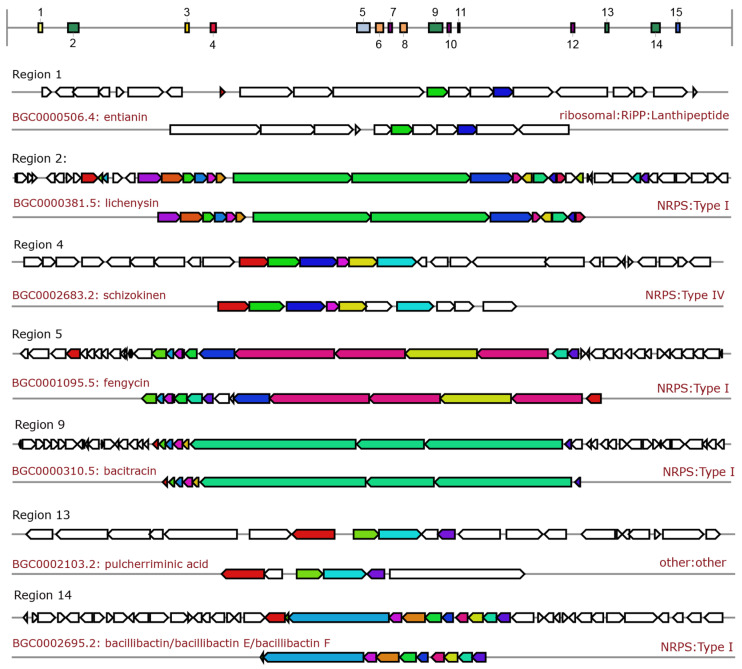
Comparative KnownClusterBlast alignment of major BGCs predicted in strain ITBMC36 using antiSMASH v8.0 (accessed on 10 January 2026). Predicted secondary metabolite biosynthetic gene cluster regions are shown based on antiSMASH analysis. For each region, the upper track represents the ITBMC36 query BGC and the lower tracks show similar reference BGCs identified by ClusterBlast/MIBiG comparison. Genes shown in the same color indicate homologous genes or mutual BLAST hits between the query region and reference BGCs.

Genome mining with antiSMASH predicted 15 biosynthetic gene clusters in ITBMC36, compared with 13–14 clusters in the other *B. paralicheniformis* genomes analyzed using the same workflow. High-confidence clusters included those associated with bacitracin, fengycin, lichenysin, and bacillibactin, together with additional RiPP-, terpene-, and PKS-related loci (Figure 13; Table 4). One T1PKS cluster, approximately 45.7 kb in length, appeared to be unique to ITBMC36 among the genomes compared. These biosynthetic loci suggest that the strain may possess additional traits related to ecological fitness and strain-level differentiation.

Several predicted BGCs encode products commonly associated with antagonism or environmental fitness (e.g., bacitracin/fengycin) and surface-active compounds (e.g., lichenysin). While the chemical products and their roles in mineralization were not experimentally determined here, these loci provide markers of strain-level differentiation and targets for future metabolomic validation.

#### 3.5.6. Comparative Genome Analysis

**Figure 14:**
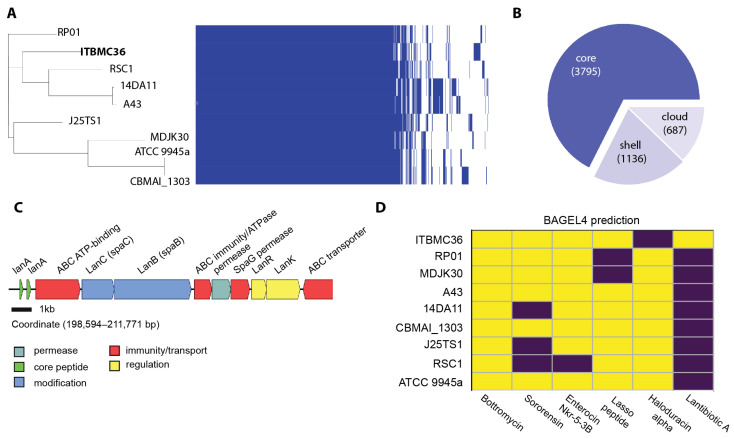
Pangenome-guided discovery of a subtilin-like class I lanthipeptide (spa) biosynthetic locus uniquely encoded by *B. paralicheniformis* ITBMC36. (**A**) Presence/absence matrix of pangenome gene families across nine *B. paralicheniformis* genomes, with strains clustered by similarity in gene content. (**B**) Pangenome partitioning into core (present in 8–9 strains), shell (present in 2–7 strains), and cloud (present in only singleton/strain-specific) gene families. (**C**) Organization of the Subtilin-like lantibiotic (lanA) locus in ITBMC36. (**D**) Heatmap summarizing bacteriocin/RiPP classes predicted across the nine genomes (BAGEL4 output). Yellow indicates presence and purple indicates absence.

Pangenome analysis of ITBMC36 together with eight publicly available *B. paralicheniformis* genomes (Table 5) identified 5617 genes across the nine strains, including 3795 core genes, 1136 shell genes, and 687 cloud genes (Figure 14A,B). A total of 128 gene families were uniquely present in ITBMC36 relative to the comparison set. Among these, functional annotation highlighted a subtilin biosynthesis dehydratase/cyclase-associated signature, including a LanC-like enzyme annotated as *SpaC* (GMDLPC_00236) and LanB-like enzyme (GMDLPC_00237).

Inspection of this genomic region identified a cluster (198,594–211,771 bp) with the canonical organization of a class I lanthipeptide biosynthetic system (Figure 14C; Table 6). The locus includes a putative precursor peptide (GMDLPC_00234; lanthipeptideA predicted by RiPPMiner), an ABC transporter (GMDLPC_00235), LanC-like cyclase (GMDLPC_00236), LanB-like dehydratase (GMDLPC_00237). Additional genes within the locus are predicted to mediate export and self-protection, including a lantibiotic protection ATPase (GMDLPC_00238) and membrane-associated proteins (GMDLPC_00239–GMDLPC_00240, including *SpaG*) implicated in secretion and/or immunity. A two-component regulatory system is also present (response regulator-LanR: GMDLPC_0024 and histidine kinase-LanK: GMDLPC_00242). Consistent with this, BAGEL4 predicted this lanthipeptide class only in ITBMC36 and not in the comparator genomes (Figure 14D). These results indicate that ITBMC36 carries a distinctive accessory biosynthetic locus that may contribute to its strain-specific functional profile.

## 4. Discussion

This study provides an integrated phenotypic, mineralogical, and genome-based characterization of *Bacillus paralicheniformis* ITBMC36 as a candidate bacterium for MICP-based crack repair in cementitious materials. Most previous work in this field has focused on model organisms such as *S. pasteurii* and *B. subtilis* [4,7,9,10,11,12]. By comparison, *B. paralicheniformis* has received far less attention in the context of biomineralization. At least one previous study has reported calcite precipitation by a *B. paralicheniformis* isolate [17], indicating that the species is not entirely new in the MICP literature. However, studies that combine biomineralization assays, mineral characterization, application testing in cementitious materials, and complete genome analysis for this species remain limited. In that context, the present study extends earlier observations by linking the MICP phenotype of *B. paralicheniformis* to a high-quality complete genome and a laboratory crack-healing assay.

A notable result of this study is the strong mineral precipitation capacity of ITBMC36. The strain produced 26.73 ± 0.81 g/L precipitate in B4 medium, which is higher than yields reported for several previously described calcite-precipitating isolates under their respective assay conditions. For example, a study from Mindanao (Philippines) reported multiple calcite-precipitating bacteria, including a *B. paralicheniformis* strain with a yield of ~10.5 g CaCO_3_/L and other isolates ranging from ~10–19 g CaCO_3_/L [17]. Additional work reported precipitation yields of ~8.5 g CaCO_3_/L for *Lysinibacillus* isolates from mangrove environments in Thailand [42] and ~10–12.4 g CaCO_3_/L for several *Bacillus* isolates from Vung Tau, Vietnam [43]. While direct comparison across studies should be interpreted cautiously because precipitation yield depends strongly on medium composition, incubation time, calcium source, and quantification methods, these published ranges provide context suggesting that ITBMC36 is a comparatively strong biomineralizer under the conditions tested here.

In addition, the precipitate produced by ITBMC36 was dominated by calcite, with minor vaterite and aragonite, which is consistent with many bacterial mineralization systems in which metastable phases may form initially but calcite becomes dominant over time because of its higher thermodynamic stability [3,5]. This mineralogical profile is favorable for crack filling because calcite is generally considered the most stable and desirable CaCO_3_ polymorph in cementitious applications.

The enzymatic data support this mineralization phenotype. Under the assay conditions used here, urease activity in ITBMC36 exceeded that of the reference strain *S. pasteurii* DSM33, whereas carbonic anhydrase activity was comparable between the two strains, with measured values of 1.62 and 1.63 U/mL, respectively (see Figure 1). Because *S. pasteurii* is one of the best studied ureolytic bacteria for MICP [7,9], this comparison is useful in placing ITBMC36 within the broader field. The results suggest that ITBMC36 has a carbonate-generating capacity that is at least competitive with a conventional reference strain. In addition, this level of activity aligns with previous prior reports that indicate CA activities of 1.79 U/mL, 1.42 U/mL, and 1.55 U/mL in the biomineralizing strains *Bacillus licheniformis*, *Bacillus toyonensis*, and *Bacillus pumilus*, respectively [44]. The results suggest that ITBMC36 has a carbonate-generating capacity that is at least competitive with a conventional reference strain.

From an application perspective, ITBMC36 promoted progressive closure of artificial mortar microcracks and reduced water absorption during the early soaking period. These findings are in line with previous reports showing that MICP treatments can reduce permeability, decrease capillary water uptake, and improve crack sealing in cementitious materials. Early surface-treatment and crack-repair studies demonstrated that bacterial carbonate precipitation can decrease capillary absorption and limit ingress of aggressive ions, supporting the use of water transport metrics as functional endpoints for self-healing performance [45,46]. Other studies also emphasized that the healing outcomes depend on crack width, curing, and delivery strategies across different cementitious systems [47,48]. More recent mortar-focused work similarly combines image-based crack closure with permeability/absorption measurements to quantify healing efficiency under controlled regimes [49]. Together, these studies provide useful context for interpreting the crack-area restoration and water-absorption trends observed for ITBMC36. Notably, crack closure was observed after a single application of the bacterial suspension rather than repeated reapplication, suggesting that ITBMC36 can deposit and retain mineralizing components within the crack space to support continued CaCO_3_ accumulation over time.

The genome sequence provides a mechanistic framework for interpreting the observed phenotype. ITBMC36 contains a complete clustered urease locus, including structural genes, accessory genes required for nickel-dependent enzyme maturation, and nearby transport functions related to substrate and cofactor uptake. This organization is fully consistent with the strong urease activity and precipitation phenotype observed experimentally. Previous genetic work in *B. subtilis* has shown that enhancing urease-associated functions can substantially improve bacteria-induced calcite precipitation [4], and the genomic architecture of ITBMC36 fits well with that broader mechanistic model. Compared with the double-operon urease organization reported for *S. pasteurii* [50], ITBMC36 carries a single contiguous urease region, similar to that conserved in other *B. paralicheniformis* genomes [51]. Although operon structure alone does not directly predict mineralization efficiency, this comparison highlights that distinct genomic organizations may underlie similar MICP phenotypes in different taxa.

Beyond ureolysis, ITBMC36 encodes five predicted carbonic anhydrases, including β- and γ-class enzymes. This comparatively broad CA repertoire may provide flexibility in inorganic carbon interconversion and help sustain carbonate supply under changing intracellular or extracellular conditions. Increasing mechanistic evidence indicates that efficient biomineralization is often multifactorial and can involve carbonic anhydrases, extracellular polymers, ion transport, and biofilm-associated processes [3,4,5,6]. In ITBMC36, the coexistence of urease, multiple CAs, and biofilm-associated functions supports this integrated view and suggests that mineralization may reflect both alkalinization and matrix-assisted nucleation/retention effects [52].

The extracellular matrix-related features identified in ITBMC36 also align well with prior work highlighting the role of EPS and biofilm structure in bacterial calcite precipitation. Exopolysaccharides and capsular polysaccharides can influence calcium carbonate nucleation and crystal morphology [5,6]. In ITBMC36, genome mining identified a near-complete EPS-associated region, the *tapA*–*sipW*–*tasA* operon for amyloid-like matrix fibers, and a *pgsE*–*pgsA*–*pgsC*–*pgsB* locus for poly-γ-glutamic acid biosynthesis. Together, these features suggest that ITBMC36 can generate a chemically reactive extracellular scaffold that may bind calcium, localize cells within the crack, and favor surface-associated mineral growth. This interpretation is consistent with SEM observations showing close association between bacterial cells and mineral material, and it may help explain why crack filling progressed over time after a single application.

The genome further indicates substantial potential for persistence under cementitious conditions. ITBMC36 carries multiple Na^+^/H^+^ antiporters, a complete Mrp/Mnh-type system, oxidative stress defense genes, osmoprotection systems, and an extensive sporulation and germination machinery. These traits are relevant because concrete environments impose high pH, osmotic stress, and fluctuating nutrient availability. Similar survival-associated features have been considered important in the selection or engineering of *Bacillus*-based MICP agents [4,9,11], and the complete genome enables these traits to be documented here in a defined strain background.

Finally, comparative pangenome analysis against eight *B. paralicheniformis* genomes identified a subtilin-like class I lanthipeptide (*spa*) cluster as a distinctive accessory feature of ITBMC36. Core metabolic and sporulation genes were conserved across the species, whereas the specialized lanthipeptide locus (GMDLPC_00234–GMDLPC_00242) was absent in all eight comparator genomes. This locus includes the hallmark modification enzymes SpaB (LanB-like dehydratase) and SpaC (LanC-like cyclase), supporting maturation of the predicted lanthipeptide precursor. The presence of this strain-specific antimicrobial cluster may contribute to ecological competitiveness in microbial communities; however, confirming expression and antimicrobial activity will require targeted experimental validation.

### Limitations and Future Directions

This study used a controlled laboratory mortar model with drying-induced microcracks; therefore, the results represent a proof-of-concept evaluation rather than a full engineering validation of self-healing under load-induced cracking and field exposure. Drying-induced cracks are useful for preliminary testing because they enable relatively consistent crack widths and controlled comparisons among treatments; however, they may differ from load-induced cracks in morphology, internal damage distribution, and transport behavior. Future work should evaluate ITBMC36 in load-induced crack systems and under relevant exposure cycles (e.g., wet–dry and chloride environments). In addition to crack area closure and water absorption, durability-oriented metrics such as permeability/sorptivity, chloride penetration, and recovery of mechanical properties should be included [44,45,46]. In parallel, delivery strategies that improve retention and longevity (e.g., encapsulation or carrier-assisted/embedded formats) should be compared with the external treatment approach used here. Finally, because ureolysis can generate ammonium, optimization of urea/calcium dosing and assessment of potential by-products will be important for field-scale implementation.

## 5. Conclusions

This study identified strain ITBMC36 as *Bacillus paralicheniformis* and demonstrated its strong capacity for calcium carbonate precipitation and crack sealing in a laboratory mortar model. The strain produced calcite-dominant precipitates, showed substantial urease and carbonic anhydrase activities, and promoted progressive closure of artificial microcracks with reduced early water absorption. Complete genome analysis revealed a set of traits consistent with biomineralization and survival in cementitious environments, including a full urease system, multiple carbonic anhydrases, extracellular matrix-associated loci, and genes related to pH homeostasis, stress response, and sporulation. Overall, ITBMC36 represents a promising locally sourced bacterium for MICP-based crack repair, and future work should focus on optimizing delivery strategies and evaluating performance under more application-relevant conditions.

## Figures and Tables

**Table 1:** Carbohydrate utilization and enzymatic activities of ITBMC36 determined using API 50 CH and API 20 E assays.

Carbon Source Utilization (API 50 CH Strip Test)				Enzymes (API 20 E Strip Test)	
Test	Result *	Test	Result *	Test	Result *
0	−	Esculin	+	ONPG (beta-galactosidase)	+
Glycerol	+	Salicin	+		
Erythritol	−	Cellobiose	+	ADH (Arginine dihydrolase)	+
D-Arabinose	−	Maltose	+		
L-Arabinose	+	Lactose	+	LDC (Lysine decarboxylase)	−
Ribose	+	Melibiose	−		
D-Xylose	+	Sucrose	+	ODC (Ornithine decarboxylase)	−
L-Xylose	−	Trehalose	+		
Adonitol	−	Inulin	−	Citrate utilization	−
Methyl xyloside	−	Melizitose	−	H_2_S production	−
Galactose	+	D-raffinose	−	Urease	+
D-Glucose	+	Starch	+	Indole production	−
D-Fructose	+	Glycogen	+	TDA (Tryptophane deaminase)	−
D-Mannose	+	Xylitol	−		
Sorbose	−	Gentibiose	−	Voges-Prokauer (Acetoin production)	+
Rhamnose	−	Turanose	+		
Dulcitol	−	Lyxose	−	Gelatinase	+
Inositol	−	Tagatose	+	Other tests	
Mannitol	+	D-fucose	−	Nitrate reductase	+
Sorbitol	+	L-fucose	−	Catalase	+
Methyl-D-mannoside	−	D-Arabitol	−	Oxidase	+
Methyl-D-glucoside	+	L-Arabitol	−	Gram staining	+
N-acetyl-glucosamine	−	Gluconate	−	Endospore forming	+
Amygdalin	+	2, Keto-gluconate	−		
Arbutin	+	5, Keto-gluconate	−	* *Legend: (−) negative; (+) positive*	

**Table 2:** Genomic features of *Bacillus paralicheniformis* ITBMC36.

Attribute	Value
Genome Size (bp)	4,410,549
GC Content (%)	45.8
Number of Contigs	1
Plasmids	0
Coding Sequences (CDSs)	4647
tRNA Genes	81
rRNA Genes	24
CheckM2 Contamination (%)	0.22
BUSCO v5 Completeness (%)	99.3
GenBank Accession	CP196957.1

**Table 3:** Key genes per functional module associated with ureolysis-driven MICP and cementitious-environment fitness in *Bacillus paralicheniformis* ITBMC36.

Module	Key Genes	Bakta Locus Tag(s) GMDLPC_
Urease (structural)	*ureA*–*ureB*–*ureC*–*ureE*–*ureF*–*ureG*–*ureD*	02033, 02034, 02035, 02037, 02038, 02039, 02040
Urea/Ni-linked component in urease region	*ureH*	02041
Urea uptake	*urt* (urea transporter)	02032
Carbonic anhydrase	*paaY*, *ybcF*, *paaY*, *cynT*, *yvdA*	00290, 00897, 03350, 03371, 03821
γ-PGA biosynthesis (pgs/cap)	*pgsE*–*pgsA*–*pgsC*–*pgsB*	03948, 03949, 03950, 03951
EPS/biofilm scaffold	*tasA* (matrix protein)	02740
EPS-associated genes (examples)	*epsA*/*D*/*E*/*F*/*G*/*I*/*J*/*K*/*L*/*M*/*N*	03799, 03796, 03795, 03794, 03793, 03791, 03790, 03789, 03788, 03787, 03786
Biofilm surface layer	*bslA*, *bslB*(x2)	04112, 04214, 04215
pH homeostasis/alkali tolerance (Mrp/Mnh/Nha-type)	*mnhB*, *mnhE*, *hyfB*, *yuiF* (Na^+^/H^+^ antiporter subunits)	03499, 03503, 03502, 03538
pH homeostasis (Na^+^/H^+^ antiporters)	*nhaC* (multiple copies)	00551, 01102, 04238
General stress regulation	*sigB* + regulators (*rsbU*, *rsbV*, *rsbW*)	*sigB*: 00581; *rsb*: 00578/00582, 00579, 00577/00580
Sporulation regulators	*spo0A*; *sigF*/*sigE*/*sigG*/*sigK*	02697; 02591; 01792; 01793; 02875
Oxidative stress defense	*katA*/*katE*; *sodA*; *ahpC*/*ahpF*	*katA*: 04226; katE: 04225; sodA: 02355; ahpC: 04385; ahpF: 04386
Osmoprotection (compatible solutes)	*proVWX* (glycine betaine/proline transporter)	02730; 02731; 02732
Osmoprotection (ABC osmoprotectant transport)	*opuBB*/*opuCC*	*opuBB*: 03753 (also 03755); *opuCC*: 03754

**Table 4:** AntiSMASH-predicted biosynthetic gene clusters in *B. paralicheniformis* ITBMC36 with no close match to known reference clusters.

Region	Type	Location (Nucleotides)	Most Similar Known Cluster
3	azole-containing-RiPP protocluster	1,084,992–1,106,926	unknown
6	T1PKS	2,243,553–2,289,294	unknown
7	Terpene	2,320,729–2,342,618	unknown
8	T3PKS	2,392,065–2,433,162	unknown
10	Terpene precursor	2,679,093–2,699,983	unknown
11	RiPP-like	2,743,468–2,753,812	unknown
12	terpene-precursor	3,431,184–3,452,095	unknown
15	lassopeptide	4,070,153–4,092,886	unknown

**Table 5:** Strains used in Pan Genome analysis.

	*Bacillus paralicheniformis*	NCBI Accession ID	Source	Country
1	ITBMC36	CP196957.1	limestone	Vietnam
2	A4-3	CP043501.1	Tomato	Republic of Korea
3	14DA11	CP023168.1	Fermented Soybean	Republic of Korea
4	RP01	CP118744.1	Root surface	China
5	CBMAI 1303	CP033389.1	Soil	Brazil
6	MDJK30	CP020352.1	Rhizosphere of Peony	China
7	RSC-1	AP023088.1	Red sea water	Saudi Arabia
8	ATCC 9945a	CP005965.1	unknown	USA
9	J25TS1	AP025339.1	Honey	Japan

**Table 6:** Subtilin-like (lanthipeptideA) cluster gene list in the ITBMC36 genome. The plus (+) and minus (−) signs indicate that the corresponding genes are located on the positive or negative DNA strand, respectively.

Locus Tag	Start	Stop	Strand	Gene
GMDLPC_00234	198,594	198,731	+	*Lan A*, lantibiotic precursor peptide
GMDLPC_00235	199,249	201,039	+	ABC transporter ATP-binding
GMDLPC_00236	201,105	202,418	+	*spaC*, Subtilin biosynthesis protein
GMDLPC_00237	202,441	205,518	+	*spaB*, Subtilin biosynthesis protein
GMDLPC_00238	205,645	206,349	+	Lantibiotic protection ABC ATPase (immunity)
GMDLPC_00239	206,361	207,119	+	Permease
GMDLPC_00240	207,119	207,886	+	*spaG*, Lantibiotic ABC transporter permease
GMDLPC_00241	207,900	208,568	+	*Lan R*, response regulator (TCS)
GMDLPC_00242	208,559	209,917	+	*Lan K*, histidine kinase
GMDLPC_00243	210,035	211,771	−	*mdlB*, ABC transporter

## Data Availability

The complete, single-chromosome genome sequence of Bacillus paralicheniformis ITBMC36 is available in the NCBI GenBank database under accession number CP196957.1 at https://www.ncbi.nlm.nih.gov/nuccore/CP196957.1 (accessed on 12 August 2025). The raw Illumina and Oxford Nanopore sequencing reads used for the hybrid assembly, along with the genome annotation, have been deposited in the NCBI Sequence Read Archive (SRA) SRR36035439 and SRR36035440, respectively. The detailed project data are accessible under the umbrella BioProject accession number PRJNA1364983 and BioSample accession number SAMN53263907. The specific strain isolate is registered in the Institute of Life Science, bacterial database under accession ITBMC36. The genome annotation generated using Bakta, including GFF3 and GenBank-format files, is provided as Supplementary Data. All locus tags reported in this study correspond to the Bakta annotation. Orthology-based functional annotations generated with eggNOG-mapper and curated gene lists used for MICP analysis are also available as Supplementary Tables.

## Supplementary Materials

The following supporting information can be downloaded at: https://www.mdpi.com/article/10.3390/microorganisms14071437/s1; Table S1: Complete Bakta annotation file for ITBMC36, including gene identifiers, genomic coordinates, product names, and functional notes (TSV); Table S2: Complete inventory of MICP-related genes in *Bacillus paralicheniformis* ITBMC36 used for gene counting in Figure 7 (Bakta and eggNOG-based annotations with genomic coordinates and locus tags); Table S3: BLASTp validation of predicted carbonic anhydrase-related proteins and urease operon components encoded in the genome of *Bacillus paralicheniformis* ITBMC36.

## Abbreviations

The following abbreviations are used in this manuscript:

ANI	Average Nucleotide Identity
CA	Carbonic Anhydrase
BGC	Biosynthetic Gene Cluster
dDDH	Digital DNA-DNA Hybridization
EPS	Exopolysaccharide
MICP	Microbially Induced Calcite Precipitation
NRPS	Nonribosomal Peptide Synthetase
RiPP	Ribosomally synthesized and post-translationally modified peptides
SEM	Scanning Electron Microscopy
XRD	X-ray Diffraction
